# Effects of Ionic Liquids on Metalloproteins

**DOI:** 10.3390/molecules26020514

**Published:** 2021-01-19

**Authors:** Aashka Y. Patel, Keertana S. Jonnalagadda, Nicholas Paradis, Timothy D. Vaden, Chun Wu, Gregory A. Caputo

**Affiliations:** 1 Department of Chemistry and Biochemistry, Rowan University, Glassboro, NJ 08028, USA; patela87@students.rowan.edu (A.Y.P.); paradi84@students.rowan.edu (N.P.); vadent@rowan.edu (T.D.V.); wuc@rowan.edu (C.W.); 2 Department of Biological Sciences, Rowan University, Glassboro, NJ 08028, USA; jonnal56@students.rowan.edu; 3 Department of Molecular and Cellular Biosciences, Rowan University, Glassboro, NJ 08028, USA

**Keywords:** ionic liquids, metalloproteins, protein denaturation, protein folding

## Abstract

In the past decade, innovative protein therapies and bio-similar industries have grown rapidly. Additionally, ionic liquids (ILs) have been an area of great interest and rapid development in industrial processes over a similar timeline. Therefore, there is a pressing need to understand the structure and function of proteins in novel environments with ILs. Understanding the short-term and long-term stability of protein molecules in IL formulations will be key to using ILs for protein technologies. Similarly, ILs have been investigated as part of therapeutic delivery systems and implicated in numerous studies in which ILs impact the activity and/or stability of protein molecules. Notably, many of the proteins used in industrial applications are involved in redox chemistry, and thus often contain metal ions or metal-associated cofactors. In this review article, we focus on the current understanding of protein structure-function relationship in the presence of ILs, specifically focusing on the effect of ILs on metal containing proteins.

## 1. Introduction

Proteins are long chain polymers of amino acids connected by peptide bonds. These polypeptide chains are interlinked with hydrogen bonding, which leads to the formation of secondary structures in proteins and subsequent further organization of these secondary structure elements form tertiary structures [1]. Protein function is governed by the specific three-dimensional structure the protein adopts by arranging the appropriate functional groups in the proper orientation. Proteins are involved in multiple processes in the living cell and are located on the extracellular surface, intracellular region, and in the cell membrane [2]. Some examples of proteins that are commonly found in biological systems are hormones, antibodies, enzymes (biological catalysts), transporters, and receptors [3]. Because of these biological functions, proteins are also used as components of industrial processes and as therapeutic agents using specific formulations [4]. Industrial processes utilize a variety of proteins such as metalloproteases, laccases, cellulases, lipases, phosphatases, and amylases for numerous applications [5]. Therapeutically, proteins such as immunoglobulins, erythropoietin, interferons, insulin, and anti-clotting proteins are widely used in the clinic [5]. Depending on the structure of the protein, they are only stable in specific physiochemical environments, and therefore it is important to evaluate the effects of various physical and chemical conditions for developing a robust formulation [6].

In some cases, protein structures are associated with metal ions, including, but not limited to, Ca^2+^, Mg^2+^ Cu^2+^, Fe^2+^, and Zn^2+^, and this class of proteins is referred to as metalloproteins [7]. Nearly 50% of the existing proteins in nature are metalloproteins [7]. Metal ions within metalloproteins play a key functional role in many biological redox processes and can provide structural stability to the protein [8]. Metal ions within these proteins play important roles not only in catalyzing biological processes but are also involved in binding interactions with organic and inorganic molecules [9]. Examples of processes that metalloproteins are involved with include the process of neuronal signal transmission, oxygen transport to and from the lungs, control of numerous redox processes, and nitrogen fixation [10]. Well known examples of metalloproteins include many electron transfer proteins (cytochrome b5, azurin, and [4Fe4S]-ferredoxin), oxygen binding proteins (myoglobin and hemoglobin), as well as multiple enzymes such as oxidases (methane monooxygenase, heme-coper oxidase, cytochrome P450, and laccase), peroxidases (horseradish peroxidase), hydrolases (carbonic anhydrase), hydrogenases ([FeFe]-hydrogenase), and reductases (copper nitrite reductase, nitric oxide reductase, sulfite reductase) [11,12,13]. In addition to the metals listed above, many proteins have been demonstrated to bind and utilize “trace” metals, or those that are not found in high concentrations in biological organisms. These trace metals, and the metalloproteins that utilize them, are an area of renewed interest as a result of improving methods to identify and characterize the metals and proteins [14].

The metals that bind to the protein are dependent on the metals available to the organism in general, and the protein’s ability to functionally adapt to the metals available. For example, many proteins in plants use the available iron from their environment, while organisms in the oceans often use copper instead of iron more frequently due to the scarcity of iron in the oceans [7]. Ion channels in the cell membrane are utilized to import these environmentally derived ions into the cytoplasm for further use by the cell. These ion channels can be selective for one or two ion species, or can be more promiscuous, allowing multiple different species through the channel [15]. Once acquired, the location of the metal within the protein is key as it should not preferentially interact with the surrounding environment [13]. The structure of a metalloprotein is partially dependent on the metal; however, this structure can often be slightly modulated to accept a variety of similarly sized and charged metal ions [16,17]. This is a complex interplay between the folded protein and the binding pocket for the metal. The protein can often fold into a similar structure in the absence of the metal, referred to as the apo-form. With the metal present, there are additional intermolecular contacts formed that stabilize the structure, known as the holo-form. Importantly, metal atoms of similar size/charge/valence may interact with the same binding site, although the protein structure is usually most stable with native metal ion ligand. The native 3D structure of the metalloprotein allows the interaction of amino acid side chains with the appropriate type and number of metal ligands. This orientation promotes the correct metal-amino acid geometry facilitating the functional role and reactivity of the metal ions [13]. Protein folding is important for protein stability, and each polypeptide can adopt different three-dimensional conformations depending on the microenvironment in which it is being held [18,19]. Changes in the surrounding microenvironments may lead to the addition or removal of the metal ions from the protein, which can impact the stability of the protein [13].

Importantly, proteins are not the only biomolecules that interact with metal ions in nature. Small organic molecules, carbohydrates, and nucleic acid interactions with metal ions have all been well established in the literature. Again, in these cases, the metal ions can be structural and/or catalytic in functionality. Examples include the structural bridging of alginate chains by Ca^2+^ [20], Mg^2+^ bridging and charge stabilization of the bacterial lipopolysaccharides [21], stabilization and structural modification of DNA and RNA by numerous monovalent and divalent cations [21,22,23], and metal-mediated catalysis by nucleic acids [24,25]. For readers interested in a comprehensive review of metal ion interactions with biomolecules, we suggest the recent reviews by Shchreiber and coworkers and/or by Bechtold and coworkers [26,27].

### 1.1. Protein Folding/Unfolding

Protein folding is the process by which the primary amino acid chain adopts an active 3D structure that is capable of carrying out the evolved function. The investigation of how proteins fold and unfold along with the forces that govern these processes has been an area of intense study for >50 years [28,29]. In nature, the folding process occurs in the cell and is often aided by chaperone proteins or very specialized local environments such as the interior of the transcolon. However, it can occur in vitro as well, which is more dependent on the specific protein sequence and the environment [30]. Protein folding is achieved by the 3D rearrangement of a linear polypeptide chain, driven through Van der Waals interactions, hydrogen bonding, hydrophobic burial, and electrostatic interactions. All of these interactions occur between protein moieties, ligands, cofactors, and solvent molecules [28,29]. This allows for amino acid functional groups to be brought together, enabling chemical processes to occur per the specific protein function [30,31]. Most often, the key to the proper folding of proteins lies in their amino acid sequence [32]. The initial steps of this process often involve the burial of hydrophobic groups in a collapsed form, followed by the formation of secondary structures driven through electrostatics and hydrogen bonding. Regardless of whether or not the protein can spontaneously fold, there are thermodynamic and kinetic constraints that govern the folding process. In terms of thermodynamics, the protein must be able to fold into the native conformation that is stable under the environmental conditions where the protein must carry out the evolved function. In cells, proteins are often only marginally stable, which allows for effective degradation of these molecules when needed [33]. In terms of kinetics, a denatured or unfolded polypeptide chain must be able to achieve the native conformation state in a period of time that is reasonable within the constraints of cellular function [34]. The secondary structure, alpha helices, and beta sheets, and the tertiary structure are also dependent on the primary sequence, which are integral parts of the proper 3D structure allowing for proper 3D positioning of functional groups from the amino acid side chains [31,32].

When a protein unfolds or denatures, it means that the protein has lost stability in the functional 3D-structure, resulting in the protein being more flexible. This process is driven by the disruption of the bonds that drive the protein to fold, such as hydrogen bonds or electrostatics. If a protein is in its native conformation, it requires some physical or chemical interactions to initiate the unfolding process, which is usually achieved by increasing temperature or adding a chemical denaturant [35] in order to understand the thermodynamics and stability of a protein in vitro [36]. There are four main denaturation techniques that can be utilized: chemical denaturants, temperature, pressure, and force. By utilizing these various denaturants to unfold the proteins, various aspects of protein stability can be elucidated [37]. There are numerous studies which have been performed to evaluate the effect of physical stress conditions such temperature, pressure, agitation, and packing/container/closure surface, as well as the effect of chemical change in terms of pH, surfactant, inorganic salts, ILs, and co-solvents, folding and unfolding of proteins [38,39,40,41,42,43].

Chemical denaturation is a widely used approach allowing greater understanding of protein stability. One of the most common denaturants is urea, which acts by causing the disruption of nearby water-water interactions and increasing hydrogen bonds between urea molecules and the backbone of the protein. These interactions allow for increased hydrophobic solvation, which furthers the process of unfolding [44]. Another common denaturant is guanidinium, most commonly used as a hydrochloride salt (GuHCl). The exact mechanism by which GuHCl denatures proteins is still a controversy in the field, but various work has indicated hydrogen bond disruption, water-interactions, hydrophobic interactions, or backbone contacts as possible mechanisms [45,46]. There is some evidence that GuHCl is more effective at denaturing β-sheets [46,47]. In addition to these chemical denaturants, temperature, or pH changes are also used to evaluate unfolding processes in the proteins [48]. Increasing the temperature provides enough thermal energy to increase molecular motion, and a change in pH impacts the charge state of ionizable groups in the protein, which impacts the electrostatic folding forces [49].

In one study, the C12 protein underwent protein unfolding at high temperatures, influencing the rate at which it unfolds [48]. C12 is a globular protein with one domain and has been considered to be a good model for studying protein unfolding. During the unfolding process, there were disruptions in the structure of the protein core that were caused by hydrogen bond disturbances. This experiment showed that the unfolding process is an activated process since the protein would first disrupt the core protein structure and then underwent sliding movements that caused it to unfold into its transition state. Following this, the protein fully denatured with no native structure present [48]. This represents only one example of numerous reports in the literature of protein denaturation. Other well studied model systems include ribonuclease A, T4 lysozyme, myoglobin, and others [50,51,52,53,54].

In order to understand the unfolding process in a protein, several spectroscopic methods can be used, with the most common being fluorescence, absorbance, circular dichroism (CD), infrared (IR), and nuclear magnetic resonance (NMR). UV-Vis absorbance is a widely utilized analytical technique, although not all proteins have appropriate chromophores that exhibit spectroscopic changes upon unfolding [55,56,57]. One example where the UV absorbance was used was to follow the changes that happened after tyrosyl ionization; during the unfolding process of the protein in pH, the tyrosine residues, which are buried in the protein structure, are uncovered, and UV absorbance can detect the tyrosyl ionization [58]. The UV spectroscopy technique used is dependent on the protein unfolding to reveal the buried aromatic residues and make these residues exposed to the solvent, showing an increased absorbance in the 280–310 nm range due to tyrosine formation at high pH. UV spectroscopy revealed whether or not the protein was undergoing an unfolding transition event [59]. The experiment showed that the technique used on the UV measurements to analyze protein denaturation is viable and can be used to understand other proteins. Fluorescence spectroscopy is also commonly used to follow the denaturation of proteins, generally utilizing the intrinsic fluorescence of Trp residues. The emission spectrum of Trp is inherently environmentally sensitive, exhibiting a red-shift in emission maximum when moving from a less polar environment to a more polar environment [60,61,62]. Trp residues buried in the hydrophobic core of a protein will exhibit such a change in environment upon protein denaturation. These emission shifts have been widely utilized to study denaturation in proteins such as myoglobin, glycoprotein E from dengue virus, β-lactoglobulin, and [63,64]. Nuclear magnetic resonance (NMR) spectroscopy is useful in determining the protein kinetics as well as the mechanism by which the protein folds and unfolds [65]. It can give information about the unfolding and folding process of proteins based on specific residue interactions using isotopically labeled proteins. It can also provide information regarding chemical shifts, which can help determine the state at which the protein exists, unfolded or folded. It reveals information through the dynamics of the protein when it is in an unfolded conformation [66]. NMR can also be used to generate high-resolution structures of proteins and peptides, including metalloproteins [67,68,69,70,71]. Similarly, electron paramagnetic resonance (EPR) spectroscopy, which relies on the spin of unpaired electrons, is useful for the study of metalloproteins with magnetic metal centers [72,73,74,75]. Circular dichroism (CD) can be used to observe the structural changes by monitoring the disappearance of specific spectral signatures associated with α-helices and β-sheets [76,77]. It can also be used to analyze how proteins form ligand with specific molecules, such as substrates and cofactors [78,79]. A variant of traditional CD spectroscopy is known as magnetic CD (MCD). This method aligns the protein sample in a magnetic field during spectroscopic interrogation, which allows for the study of energy levels in the metal. MCD has been applied to a variety of metalloproteins, including nitrogenases, cytochrome c, and aminopeptidases [80,81,82,83,84]. Similarly, traditional infrared (IR) spectroscopy has been widely utilized to study metalloprotein structures and interactions. Standard Fourier transform IR (FTIR) can monitor secondary structures in the protein, while far-infrared spectroscopy (FIR) can be utilized to interrogate low-frequency vibrations, such as those in metal complexes [85,86]. These IR methods have been used to investigate the structure of numerous metalloproteins and peptides, including EndoIII, azurin, bovine serum albumin, and natural and designed peptides [86,87,88,89,90,91]. All of these pieces of information can together provide information regarding the stability of a protein’s structure [92].

### 1.2. Ionic Liquids (ILs)

ILs are organic salts with melting points below 100 °C. In 1992 the first IL stable in air and ambient moisture was reported [93]. After that, ILs have been developed as an alternative to organic solvents and used in many more applications. ILs are useful industrial and laboratory solvents. The molecular composition of ILs is a combination of different cations and anions that leads to countless potential ionic liquid species. ILs have a wide range of physicochemical properties, including low vapor pressure, high thermal stability, high conductivity, non-flammability, and varying degrees of biocompatibility [94]. Therefore, they could be used as reaction media for synthesis and can be recycled multiple times, which underpins the “green” reputation of these solvents [95]. ILs have the ability to act as a host and can interact with both host and guest molecules via a combination of electrostatic, hydrogen bonding, π- and van der Waals interactions [96]. The non-covalent interactions within ILs are easily broken and, therefore, are commonly used to dissolve recalcitrant materials [96]. ILs are currently being used in many different applications, including electrochemistry, energy, organic synthesis, and catalysis, as well as in biotechnology [97,98,99,100].

### 1.3. Ionic Liquid Interactions with Biomolecules

In nature, biomolecules are surrounded by charged species, including proteins, polysaccharides, nucleic acids, inorganic ions, and small organic molecules. Although proteins have evolved to function in these ion-rich environments, not all ionic species have identical effects on proteins. Specifically, there has been a significant amount of study regarding the ability of ionic species to stabilize or destabilize proteins in solution. This ranking of ions based on the effects on protein solubility, known as the Hofmeister series, is a core component of understanding protein behavior in complex ionic solutions [101,102,103]. Importantly, extensive study of the Hofmeister series has determined that the anionic component of the salt generally has a larger effect on protein solubility [101,102,103]. Mechanistically, ions in the Hofmeister series are thought to change the ordering and interactions of the bulk water around the protein rather than more direct protein interactions, which then impacts protein hydration and stability [102,104,105,106]. Numerous ILs have been studied from the context of the Hofmeister series, especially since many commercially available ILs have simple anions or cations as part of the IL pair [107,108]. These studies include direct influences of ILs on biopolymers but also more fundamental studies of IL properties in solution, including physicochemical parameters such as ion hydration number, which appears to be an important factor in IL-biomolecule interactions [102,107,109,110,111,112]. When considering the descriptions of IL-protein interactions below, the IL composition and ion placement in the Hofmeister series, when known, should be considered in the reader’s interpretations.

The unique properties of IL have made them very useful as potential solvents for protein preservation, media for enzymatic reactions, as well as applications in the field of bioconversion and protein production/purification [42,113,114]. ILs are also found to enhance the solubility of certain proteins, mainly through the prevention of aggregation [115,116,117]. Furthermore, enhanced solubility of proteins in ILs can also help achieve highly supersaturated solutions, which were successfully used as an additive in media to promote protein crystallization. ILs were shown to influence the crystallization of multiple proteins as well as improving the size of the crystal formed (helping crystal growth), quality of crystals, and enhances the reproducibility of the crystallization process [118,119]. In addition, IL/aqueous bi-phasic systems were also used for the extraction of proteins from biological fluids [116,120]. These are a few representative instances where ILs can enhance protein stability and activity. However, not all ILs are compatible with proteins. Many ILs have been shown to destabilize protein structure and activity. The physicochemical properties of ILs such as polarity, alkyl chain length, hydrophobicity, and viscosity all have different effects on protein stability [42]. Therefore, a rational selection of IL for a specific protein under investigation is necessary before using it as a solvent for that application. Furthermore, there is only limited knowledge regarding the mechanism of protein stabilization or destabilization in the presence of ILs, and therefore, research is still needed to understand how they interact with proteins based on the chemistry of ILs [121].

There has been great interest in recent years to use ILs in various industries because of the beneficial properties and the desire to stabilize protein functionality over wider ranges of reaction conditions. Specifically, how these ILs interact with biomolecules and what cation-anion combinations may impact biomolecular functions are of great interest for industrial applications. Numerous groups have studied the interactions of proteins with a wide variety of ILs, resulting in some ILs enhancing protein activity and stabilizing protein structures, with others disrupting protein structures [5,122,123]. The disruptive ILs are effectively a destabilizing agent, acting as a denaturant. Exploiting the ability of some ILs to enhance protein denaturation can yield greater insights into these protein-IL interactions. In one study, ribonuclease A was used to understand the effect of ILs on protein stability and aggregation. Ribonuclease A, a small enzyme, was examined in the presence of ILs such as choline dihydrogen phosphate ([Chol][Dhp]), 1-ethyl-3-methylimidazolium dicyanamide ([EMIM][Dca]), 1-butyl-3-methylimidazolium bromide ([BMIM]Br), and choline chloride ([Chol][Cl]). From this study, it was observed that [Chol][Dhp] promoted the stability of the native state and increases the chances of refolding, which prevents protein aggregation [124]. In another study, human serum albumin (HSA), was studied in the presence of the ILs 1-butyl-3-methylimidazolium tetrafluoroborate ([BMIM][BF_4_]) and choline dihydrogen phosphate ([Chol][Dhp]). There, [BMIM][BF_4_] was shown to induce swelling of HSA loop 1, causing it to be 0.6 nm wider compared to what it is in water, although [Chol][Dhp] was not able to impart a similar effect [125]. While this is one example, there are numerous reports in the literature comparing numerous proteins with an even greater number of ILs.

## 2. Interaction of Ionic Liquids with Metalloproteins

Due to the sheer number of unique proteins found in nature, combined with the ever-increasing number of ILs, it is unlikely there will be a set of hard and fast rules that define all IL-protein interactions. As a result, it is important to begin to focus on the interpretation and analysis by refining the types of molecules being investigated. This review focuses on understanding the impact of various ILs on metalloproteins such as laccase, myoglobin, alcohol dehydrogenase, and horseradish peroxidase (HRP).

### 2.1. Effect of ILs on Laccase

**Figure 1:**
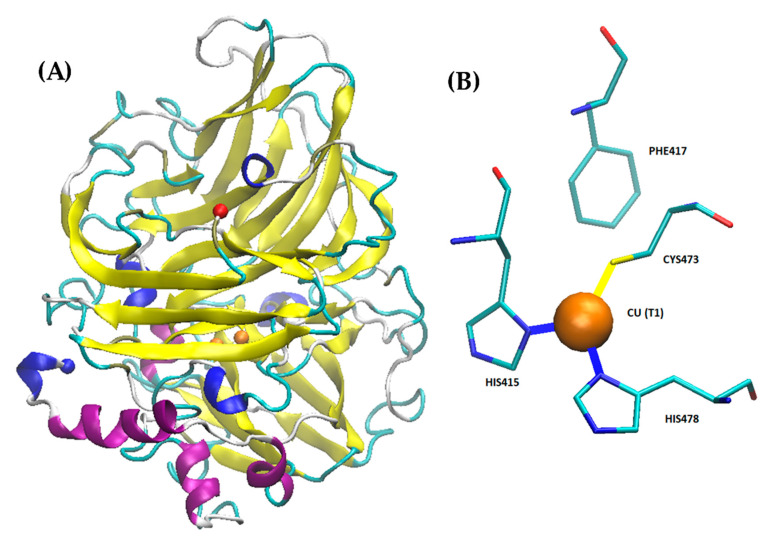
Structure of Laccase from *Trametes versicolor*. The crystal structure was solved by Choinowski and coworkers; downloaded from rcsb.org (1GYC) ([126,145]). The structure was visualized using Visual Molecular Dynamics (VMD) software. (**A**) 3D structure of laccase. The N- and C-termini are shown as red and blue spheres, respectively, while the copper ions are shown in orange (partially occluded in the structure). (**B**) structural geometry of the mono-copper site with chelating residues highlighted.

Laccase is a metal containing protein containing four copper ions in its active center [126,127]. Laccase was originally isolated from the Japanese lacquer tree *Rhus vernicifera*. After that, laccases were also found in multiple different plant sources like *Rhus succedanea*, *Acer pseudoplatanus*, *Pinus taeda*, *Populus euramericana*, *Liriodendron tulipifera*, and *Nicotiana tabacum* [128,129,130,131,132,133]. Laccases found from these sources are monomeric proteins that have molecular weights between 90–130 kDa [54]. Notably, they are also highly glycosylated, with carbohydrate content between 22–45% [134,135]. In addition to plant sources, fungi are a common source of laccase, and most fungi produce different laccase isoforms and isoenzymes. One of the most commonly studied forms of laccase is derived from the *Trametes versicolor* fungus [136,137,138,139]. The *T. versicolor* laccase contains two copper sites, a mono-copper and a tri-copper site (Figure 1). The Cu^2+^ at the mono-copper site is coordinated by two His and one Cys residue, while the Cu^2+^ atoms at the tri-copper site involve coordination of at least 3 His residues and multiple carboxyl containing residues (Asp and Glu) [127,140,141]. Recent studies show laccase is also present in bacteria, although these proteins are less well studied [142,143,144].

In nature, laccases typically oxidize phenolic compounds and reduce molecular oxygen into water after several rounds of catalysis [146]. This is typically involved in the synthesis or degradation of naturally occurring plant lignins [147]. Laccase has found utility in bioremediation of waste products from numerous industries, remediation of excess pesticides and herbicides, as well as cleaning of wastewater streams [148]. Additionally, many synthetic organic compounds can be substrates for laccase. Organic substrates of laccase are categorized into three groups: ortho-, meta-, or para-substituted compounds (all with a lone pair of electrons). In most cases of laccase, ortho-substituted compounds work as the better substrate over para- or meta-substituted compounds [144,149]. One of the most useful synthetic laccase substrates is 2,2’-Azino-bis(3-ethylbenzthiazoline-6-sulfonic acid) (ABTS), which is a colorimetric substrate allowing spectroscopic monitoring of laccase activity. ABTS was used in monitoring the oxidation of non-phenolic lignin structures, which gave the impetus to find new laccase mediators [150,151]. A particularly interesting application of laccase is in the detoxification of chlorophenol-containing wastewater, which is achieved by laccase-mediated polymerization via radical coupling [152,153]. The industrial applications of laccase, coupled with the straightforward monitoring with ABTS, have made it a very attractive system to study with ILs. A brief summary of studies that have been published on laccase with ILs can be found in Table 1.

Laccase enzymatic activity towards oxidation of ABTS was shown to increase when [MMIM][MeSO_4_] and [BMIM][MeSO_4_] were used as ILs at a concentration of 35% *v*/*v* Below this concentration ILs do not show much impact on laccase activity [158]. In another study, increased IL concentrations produce a red shift in λ_max_ for laccase fluorescence [157]. Specifically, researchers have shown that when laccase was combined with various volumetric fractions of pyrrolidinium formate ([Pyrr][F]) and morphilinium formate ([Morph][F]) ILs, they both showed a red shift in λ_max_ for laccase fluorescence [157]. However, the authors did not specifically investigate the mechanism of IL inhibition of enzymatic activity [157].

Solution pH is another parameter that is important to understand the stability of laccase in ILs. The isoelectric point (pI) of laccase is 4.6 [160,161] and based on the nature of the IL it would affect its interaction with laccase. For example, the fluorescence intensity of laccase was found to decrease in presence of the IL [TMA][TfO] more at pH 3.6 than at pH 5 [155]. On the other hand, at pH 5.8, the fluorescence intensity of laccase was found to increase in the presence of [TMA][TfO]. At pH 3.6, there is greater contribution from CF_3_SO_3_^−^ anion with respect to its interaction with the laccase interaction and as a chaotropic anion it has higher preference to bind with the protein-water interface and destabilize the enzyme (Hofmeister effect) [108,162]. However, at pH > pI (pH 5.8) the cation [TMA]^+^ is more active in terms of ordering the water structure surrounding enzyme and makes laccase more compact, resulting in increased fluorescence intensity from the greater shielding of buried Trp residues by the bulk polar aqueous milieu [155]. In another study, the effect of laccase activity in the presence of three 1-ethyl-3-methyl imidazolium ILs (with anions [MDEGSO_4_], [EtSO_4_] and [MeSO_3_]) was determined at pH 5, 7, and 9. The results show that at pH 7 and 9, the activity of laccase does not change with the addition of ILs. However, at pH 5 the laccase showed significantly reduced activity overall, but the IL-laccase samples showed a smaller loss of activity, that is, the laccase + IL mixtures performed better than laccase alone at pH 5 [163].

Above 75% (*v*/*v*) concentration of ILs like 1-ethyl-3-methyl imidazolium ILs (with anions [MDEGSO_4_], [EtSO_4_] and [MeSO_3_]) laccase precipitated under most conditions [163]. In the case of 4-methyl-N-butylpyridinium tetrafluoroborate, [4-MBP][BF_4_], laccase precipitated even at 50% (*v*/*v*) concentration. Precipitation occurs because salting out effects are promoted at high concentration. Novel formulations such as microemulsions made up of ILs, can also influence laccase activity. For example, when water-in-[BMIM][PF_6_] was used as the IL, laccase activity was found to be higher for the water-in-IL microemulsion compared to pure IL or water-saturated IL [42].

In addition, ILs can impact the biocatalytic activity of the laccase. For instance, aqueous biphasic systems containing IL cholinium dihydrogen citrate ([Chol][DHCit]) have been shown to enhance the extraction efficiency of the enzyme and increase the biocatalytic activity by 50% [164].

### 2.2. Effect of ILs on Myoglobin

**Figure 2:**
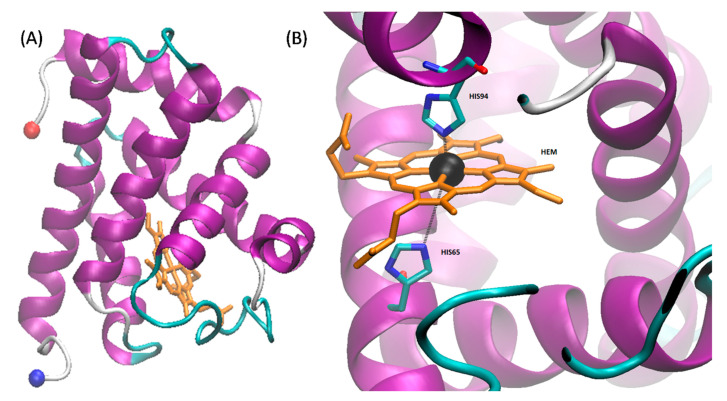
Structure of myoglobin from cardiac muscle of *Equus caballus*. The crystal structure was solved by Brayer and coworkers; downloaded from rcsb.org (1WLA) [145,173]. The structure was visualized using VMD. (**A**) 3D structure of myoglobin. The N- and C-termini are shown as red and blue spheres respectively while the heme is shown in orange (partially occluded in the structure). (**B**) Structural geometry of the heme with the iron shown in black and chelating residues highlighted.

Myoglobin (Figure 2) is a water-soluble globular protein of 150 amino acids involved in transport and storage of oxygen found in mammalian muscle tissues [165,166]. Like laccase, myoglobin is a metalloprotein having an iron atom incorporated in the heme group which together are involved in reversibly binding oxygen [167]. The heme binding site of the protein contains two His residues, one (proximal) is attached directly to the heme iron and the other (distal) is on the opposite face of the heme but does not bind the iron, instead being available for binding to O_2_. The presence of this iron imparts a reddish-brown color to the protein and yields an intense absorption band at ~409 nm [168]. The heme group is buried under a hydrophobic pocket of the myoglobin in its native folded state, however, upon unfolding, the heme group is exposed to the aqueous environment, resulting in decrease in the absorption at ~409 nm [168]. Because of these easily interrogated absorbance properties, myoglobin has been widely used as a model protein to understand folding and unfolding kinetics as a function of the varieties of conditions involving not only thermal, pH, and mechanical stress, but also a wide range of denaturants such as detergents, organic solvents, and ILs [169,170,171,172]. A brief summary of studies that have been published on myoglobin with ILs can be found in Table 2.

In one study, the results suggested that ILs containing sulfate or phosphate ions and having higher viscosity such as diethylammonium sulfate ([DEA][SO_4_]), triethylammonium sulfate ([TEA][SO_4_]), dihydrogen phosphate ([DEA][P]), triethylammonium dihydrogen phosphate ([TEA][P]), Trimethylammonium dihydrogen sulfate ([TMA][SO_4_]) and Trimethylammonium dihydrogen phosphate ([TMA][P]) improve the stability of the myoglobin [172]. On the other hand, they also reported that less viscous ILs having acetate anions such as diethylammonium acetate ([DEA][Ac]), triethylammonium acetate ([TEA][Ac]), diethylammonium and Trimethylammonium acetate ([TMA][Ac]) were shown to destabilize myoglobin structure. One hypothesis is that ILs affect the stability of a protein by altering the hydration layer (i.e., layer of water molecules around the protein). Specifically, in this case, the authors postulated that phosphate-containing ILs significantly interact with the myoglobin polypeptide chain and hence are repelled from the protein. In addition, because of these repulsions this IL also helps to provide better structure to the hydration layer, improving the stability of the protein [172]. As the acetate ions have greater affinity toward the polypeptide chain of myoglobin, they penetrate deep inside the protein structure and interact with amino acids of the polypeptide. Therefore, acetate ions present in ILs also disturb the native hydrogen bonding pattern as well as interactions of the protein with the hydration layer, resulting in protein destabilization. Further, results have indicated that anionic variation in the ILs has greater impact on stability of myoglobin compared to the cationic variations (summarized in Table 3) [172].

In work from Zhang et al. it was demonstrated that variation in the cations can also influence the stability of myoglobin [178]. They demonstrated that GuHCl-induced denaturation midpoints of myoglobin was not altered when interacted with phosphate buffer having 150 mM of various ILs differing only in their anions such as BF_4_^−^, NO_3_^−^, Cl^−^, and Br^−^, while keeping the same cation 1-butyl-3-methylimidazolium (BMIM^+^) [178]. Furthermore, they have shown that increasing length of alkyl chain of imidazolium cation in the ILs affects denaturation of the myoglobin and the denaturation midpoint were found to be [HMIM][BF_4_] < [BMIM][BF_4_] < [EMIM][BF_4_] < buffer. Additionally, hydroxy-substitution on the imidazolium cation also enhanced the denaturation of the myoglobin [178]. These differences in variation in the effect of various ILs on their capability to stabilize or destabilize the protein structure is still an unresolved question.

While some previous studies demonstrated positive or negative impact of ILs on the stability of the myoglobin, other studies demonstrated that some ILs are inert toward the stability of myoglobin. For instance, the effects of [BMIM][Cl], [EMIM][Ac], [Pyrr][BF_4_] and [TMG][Ac] was investigated [123]. The results from this study indicated that these four ILs accelerate myoglobin unfolding kinetics not only due to changes in the aqueous solution ionic strength, but also due to IL-specific interactions [123]. While, in another study, [EMIM][Ac] did not impact myoglobin stability, but the IL [BMIM][BF_4_] drastically reduced the free energy required for myoglobin unfolding and hence significantly destabilized the myoglobin structure [62].

In addition, impact of ILs on the detergent-mediated denaturation of myoglobin was also evaluated. According to one study, inclusion of a series of ILs such as 1-butyl-3-methylimidazolium chloride (BMICl), 1-ethyl-3-methylimidazolium acetate (EMIAc), and 1-butyl-3-methylimidazolium tetrafluoroborate (BMIBF_4_) in aqueous solution had negligible impact on the detergent *N*,*N*-dimethyl-*N*-dodecylglycine betaine induced denaturation and heme-loss from myoglobin [177]. In another study, the effect of alkylated imidazolium chlorides based ILs such as [EMIM][Cl], [BMIM][Cl], [HMIM][Cl], and [OMIM][Cl] was tested on unfolding of myoglobin in the presence of different detergents such as *N*,*N*-dimethyl-*N*-dodecylglycine betaine (zwitterionic; Empigen BB^®^, and EBB), tetradecyltrimethylammonium bromide (cationic; TTAB), and sodium dodecyl sulfate (anionic; SDS) [78]. It was observed that, presence of ILs does not affect the EBB- and TTAB-induced dissociation of heme; however, SDS-induced dissociation is affected by presence of ILs. Furthermore, it was found that, heme dissociation follow a cooperative process at low IL concentration, while at high IL concentration the heme dissociation occur via more complex pattern, which could be due to micellization of the ILs or their direct interactions with the myoglobin [78].

### 2.3. Effects of IL on Azurin

**Figure 3:**
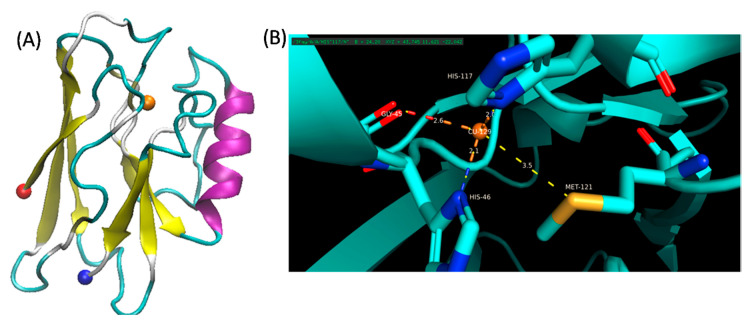
Structure of azurin from *Pseudomonas areuginosa*. The crystal structure was solved by Adman and Jensen; downloaded from rcsb.org (1AZU ([182]) [145] The structure was visualized using VMD. (**A**) 3D structure of azurin. The N- and C-termini are shown as red and blue spheres respectively while the copper is shown in orange (partially occluded in the structure). (**B**) Structural geometry of the copper shown in orange and chelating residues highlighted.

The blue copper protein, azurin, is part of the azurin-nitrate reductase redox protein complex. This protein is involved in denitrification metabolism in bacteria [87,179]. The presence of copper is necessary for protein stability. It is a small protein that can be produced from two bacterial strains—*Pseudomonas aeruginosa* and *Alcaligenes denitrificans* [180]. Azurin’s structure from *P. aeruginosa* consists of a hydrophobic alpha helix, six short beta sheets and a random-coil that allows for copper-binding [87,181,182] (Figure 3). Notably, azurin exhibits the most blue-shifted Trp emission spectrum from naturally derived proteins, arising from the single Trp residue at position 48 [183]. This is attributed to the very hydrophobic interior of the protein, which also includes the copper binding site. The Cu^2+^ is coordinated by Gly45, His46, Asn47, Cys112, Phe114, His117, and Met121 [183]. A brief summary of studies that have been published on azurin with ILs can be found in Table 4.

ILs can affect the protein structure and its stability based on several characteristics. As a protein with a mixed structure, azurin’s stability is affected differently in the presence of ILs. Recently we demonstrated 1.0 M alkyl-imidazolium chloride ILs in aqueous solutions were seen to have a variable effect on azurin; the three ILs were [BMIM][Cl], [HMIM][Cl], and [OMIM][Cl]. The difference in these three ILs are the length of the alkyl chains and hydrophobicity. Due to less hydrophobicity, [BMIM][Cl] and [HMIM][Cl] have some interactions at the surface of the protein. Furthermore, these ILs denature the secondary structure completely at a high temperature at 55 °C and the tertiary structure slowly at 65 °C. Thermodynamically, it can be observed that the ionic liquids affect that the destabilization in terms of entropy; there is an increase in entropy, as ILs increases the disorder of the unfolded protein. In general, all three ILs affect the structure of the protein by making it less rigid and flexible, while maintaining the secondary and tertiary components of the protein. [OMIM][Cl] destabilized azurin, due to a high ΔS_u_ compared to the ΔS_u_ of [BMIM]Cl and [HMIM]Cl, which was lower. Furthermore, [OMIM][Cl] was able to destabilize the protein much faster, proving that [OMIM][Cl] is stronger than the other ILs presented in this study, which were consisting of smaller alkyl chains and a decreased level of hydrophobicity [87]. It is important to note that at the concentrations tested, the [OMIM][Cl] has been shown to form micelles, These micelle structures likely impact the interactions with the protein, and can potentially form mixed structures with the protein. [87].

In a study by Fujita et al., the interaction between the hydrated IL [Chol][Dhp] and several metalloproteins, such as azurin and pseudoazurin, was investigated. The study focused on the solubility and properties of the proteins dissolved in 70 wt% [Chol][Dhp]. Specifically, in [Chol][Dhp], it was found that these proteins, when dissolved, do not have any disturbance to the active sites found in the proteins. Notably, not all proteins tested were soluble under these conditions. Among those that were soluble, the retention of structural elements was supported by spectral signatures in Raman and CD spectra. Notably, resonance raman spectra showed the peaks near 260 cm^−1^ and 400 cm^−1^ for Cu-N and Cu-S, respectively, which was consistent with the spectra for azurin in its native conformation. This indicated that the protein retained its structure and function when dissolved with the IL [184].

In another study the same IL, hydrated [Chol][Dhp], was studied to understand the interaction between the IL and azurin, specifically focusing on the redox reaction rate for azurin (dissolved in the IL) and the SAM-AuNP electrode. In the presence of this IL, it was found that the proteins were able to maintain their structure, showing long term and thermal stability. Similar to the previous study explained above, it was found that the active site of the protein was maintained in the presence of the IL using Raman spectroscopy. It was also found that electron transfer rate constant (k_s_) between azurin and the electrode in the IL (202 s^−1^) was found to be larger than that of the ammonium acetate buffer solution (44 s^−1^) and the reason for this difference could possibly be due to protein shrinkage. Both the buffer and the IL showed that electron transfer reactions were possible at a fast rate; this would mean that this fast rate would be much more stable over a broad range of temperature values and a longer time period for the IL [185].

### 2.4. Effect of ILs on Other Metal Containing Proteins

Impact of ILs has also been evaluated on other metal containing proteins such as horseradish peroxidase, alcohol dehydrogenase etc. As above, the primary purpose for these studies was to understand the how the ILs will influence folding and/or unfolding behavior of these proteins.

**Figure 4:**
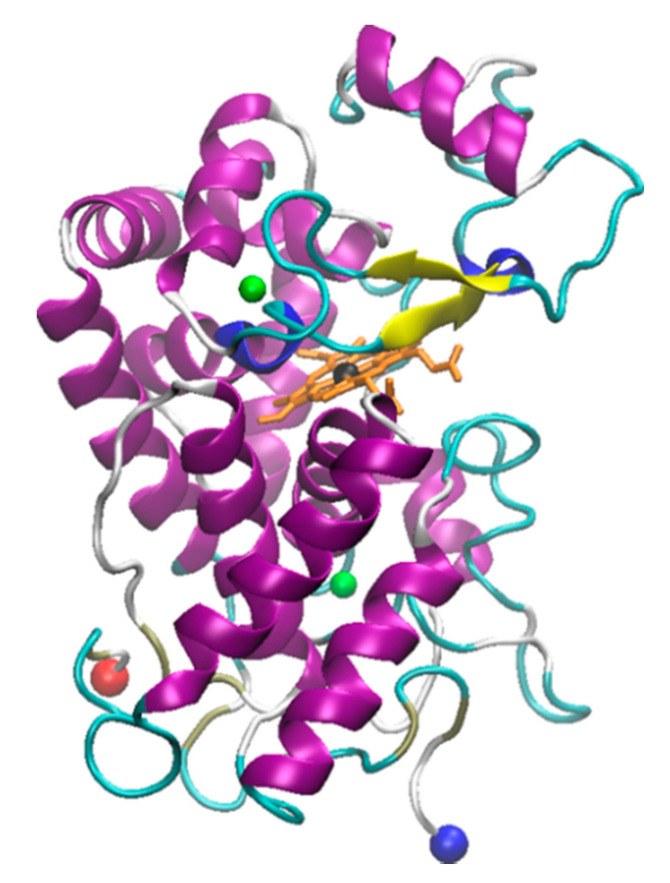
3D Structure of horseradish peroxidase from *Armoracia* *rusticana*. The crystal structure was solved by Hajdu and coworkers; downloaded from rcsb.org (1W4Y) [145,186]. The structure was visualized using VMD. The N- and C-termini are shown as red and blue spheres respectively while the calcium ions are shown in green, the heme in orange and the heme-iron in black.

Horseradish peroxidase (HRP) is an enzyme having two different metal ions namely, a ferrous ion incorporated in a heme group and a calcium ion (Figure 4). Notably, the heme-iron is directly involved in the catalytic reaction center, while the calcium is structural [186]. The effect of various ILs on activity of the HRP was evaluated using chromogenic substrates. In one study, the effect of various ILs as well as hemin and calcium cofactors were evaluated for effects on the refolding properties of HRP. This study used ILs with varying anions such as EMIM with Ac^−^, BF_4_^−^, Cl^−^, ES^−^, and TfO^−^, as well as with different alkyl chain lengths such as EMIM^+^, BMIM^+^, HMIM^+^, and OMIM^+^ [187]. Among various tested anions, Cl^−^ based ILs showed highest enzyme activity, while, among various ILs having different alkyl chain lengths, EMIM showed highest enzyme activity [187]. Notably, in the presence of IL [BMIM][PF_6_], the activity of HRP was also shown to be enhanced [188]. Moreover, HRP immobilized on a sol-gel matrix prepared from [BMIM][BF_4_] and silica was shown to have 30-fold higher activity compared to that of the enzyme immobilized on only silica gel [189].

A tailor-made IL specifically designed to work with HRP was also developed, which has the cation tetrakis(2-hydroxyethyl)ammonium 2 possible anions:Cl^−^ or [CF_3_SO_3_] [190]. This tailor-made IL has a structure similar to TRIS (buffer), possessing four hydroxyethyl moieties. Improvement in the enzyme activity was observed with the tailor-made ILs compared to that of the common, commercially available ILs such as [BMIM][Cl], [BMIM][CF_3_CO_2_], [BMIM][alanine], [BMIM][CF_3_SO_3_], [BMIM][BF_4_], and a hydrophobic IL [BMIM][PF_6_] [190]. In addition, the effect of [BMIM][Cl] and [BMIM][BF_4_] on the thermal stability of the horseradish peroxidase was also evaluated. The results of the study showed that [BMIM][BF_4_] is capable of improving the thermal stability of the horseradish peroxidase when used at a concentration of 5–10% (*v*/*v*) [191]. Furthermore, [BMIM][BF_4_] is also capable of enhancing the reaction yield and purity for the reactions converting water insoluble phenolic compounds to a novel compound 4-phenylphenol ortho dimer [2,2′-bi-(4-phenylphenol)] [192]. However, the enzymatic catalysis was sensitive to solution pH with the best catalytic activity observed with [BMIM][BF_4_] (90% *v*/*v* IL in water) at pH > 9. The enzyme activity was found to decrease as the pH was shifted toward neutral and as pH decreases further, the [BMIM][BF_4_] exerts inhibitory action on the HRP attributed to the tetrafluoroborate anion releasing fluoride ions which bind with the heme iron group [192].

**Figure 5:**
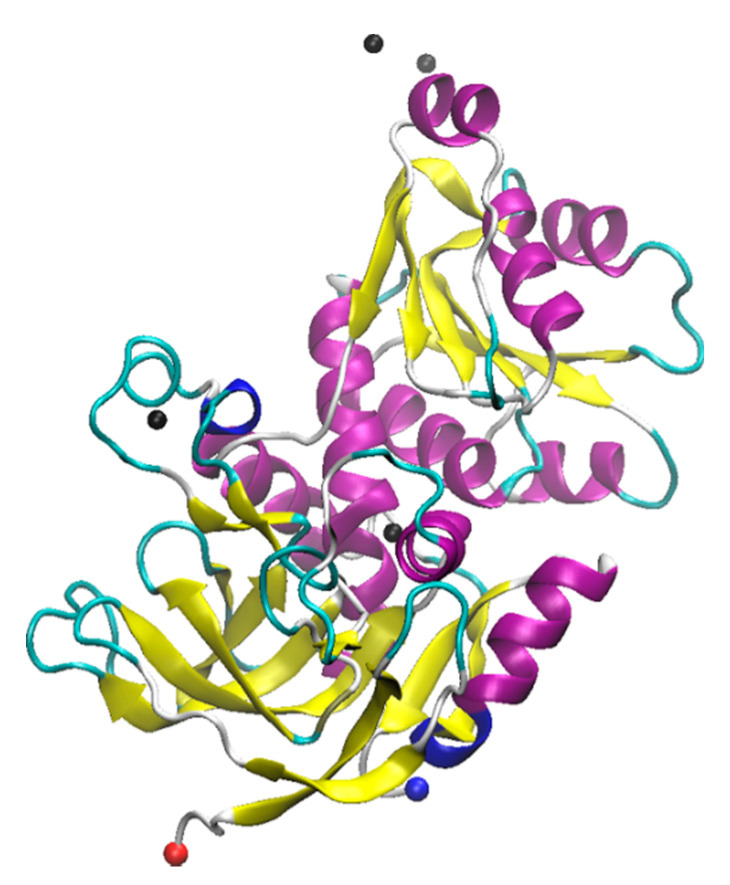
3D Structure of alcohol dehydrogenase from *Saccharomyces cerevisiae*. The crystal structure was solved by Ramaswamy and coworkers; downloaded from rcsb.org (5ENV) ([145,193]. The structure was visualized using VMD. The N- and C-termini are shown as red and blue spheres respectively while the zinc ions are shown in black. The structure represents one monomer of a homotetramer.

Alcohol dehydrogenase is another commonly studied metalloenzyme which has zinc ions in the active structure. The *Saccharomyces cerevisiae* alcohol dehydrogenase has a homotetrameric structure with each subunit having a zinc ion in the catalytic center (Figure 5) [193]. The major function of this enzyme is to carry out oxidation of alcohols using the co-substrate β-nicotinamide adenine dinucleotide (NAD^+^). This is a thoroughly studied model system that, in yeast, converts acealdehyde into ethanol along with formation of NADH and H^+^. The active site contains the Zn^2+^ atoms coordinated by Cys, His, and Glu residues [194]. In one study, the activity and stability of the yeast alcohol dehydrogenase was evaluated in solutions containing various ILs including 1-methylimidazolium chloride ([MIM][Cl]). The data showed that the order of activity enhancement was [BMIM][Cl] > [BMIM][BF_4_] > [MIm][BF_4_] ~ [MIM], while the order of stability was found to be [MIM][Cl] > [MIM][BF_4_] > control (no ILs) > [BMIM][BF_4_] > [BMIM][Cl]. The structural similarity of the cationic group of [MIM][Cl] with the adenine moiety of NAD^+^ was proposed to allow interaction with the active site and hence stabilize the enzyme at higher temperature [195]. In another study, the effect of [BMIM][PF_6_] on the yeast alcohol dehydrogenase was investigated and the data indicated a rapid decrease in the activity of the enzyme as a function of [BMIM][PF_6_] concentration [196].

The effect of variation of the anionic and cationic moieties in the ILs has also been investigated on the stability of the yeast alcohol dehydrogenases [197]. Regarding anion variation in the ILs, [EMIM] was used as a fixed cation with different anions forming [EMIM][Cl], [EMIM]Br, [EMIM][EtOSO_3_], [EMIM][TfO], [EMIM][BF_4_], [EMIM][dca], [EMIM][SCN], [EMIM][NTf_2_] [197]. In the same study, [Cl] was used as fixed anion with different cations forming NaCl, [Me_4_N][Cl], [Chol][Cl], [EMIM][Cl], [Et_4_N][Cl], [Bu_4_N][Cl], [Gdm][Cl], [BMIM][Cl]. The results of this study showed that [EMIM][Cl] and [Me_4_N][Cl] have enzyme enhancing effects on the yeast alcohol dehydrogenase, while enzyme deactivating ILs are found to have anions in the order of Br^−^ > [EtOSO_3_]^−^ > [TfO]^−^ > [BF_4_]^−^ > [dca]^−^ > [SCN]^−^ [197]. On the other hand, for variation in the cation, the enzyme deactivating order was found to be [Chol]^+^ > [EMIM]^+^ > [Et_4_N]^+^ > [Bu_4_N]^+^ > [Gdm]^+^ > [BMIM]^+^, while [EMIM][NTf_2_] was found to have strongest deactivating effect [197]. In addition, the effect of ILs on a bacterial alcohol dehydrogenase obtained from *Thermoanaerobacter brockii* (TBADH) was also investigated. Specifically, the impact of ILs such as [BMIM][Cl], [BMIM][BF_4_], [MIm][Cl] and [MIm][BF_4_] was monitored on the TBADH activity. The results showed compared to control and other ILs, the enzymatic activity and catalytic efficiency was enhanced in [BMIM][Cl] and [BMIM][BF_4_]. This study also showed that in ILs with similar anions, the activity depends on the alkyl chain length of imidazolium as well as structural similarity of cations to that of the substrate; because of this structure similarity these ILs to that of the enzyme subtract they act as an enzyme inhibitor [198]. As a result of the structural similarity of MIM ILs to that of substrate (NADP+), it was proposed that reduction in activity caused by this IL and the related [BMIM] were due to direct substrate competition rather than kosmotropic interactions with bulk water [198].

**Figure 6:**
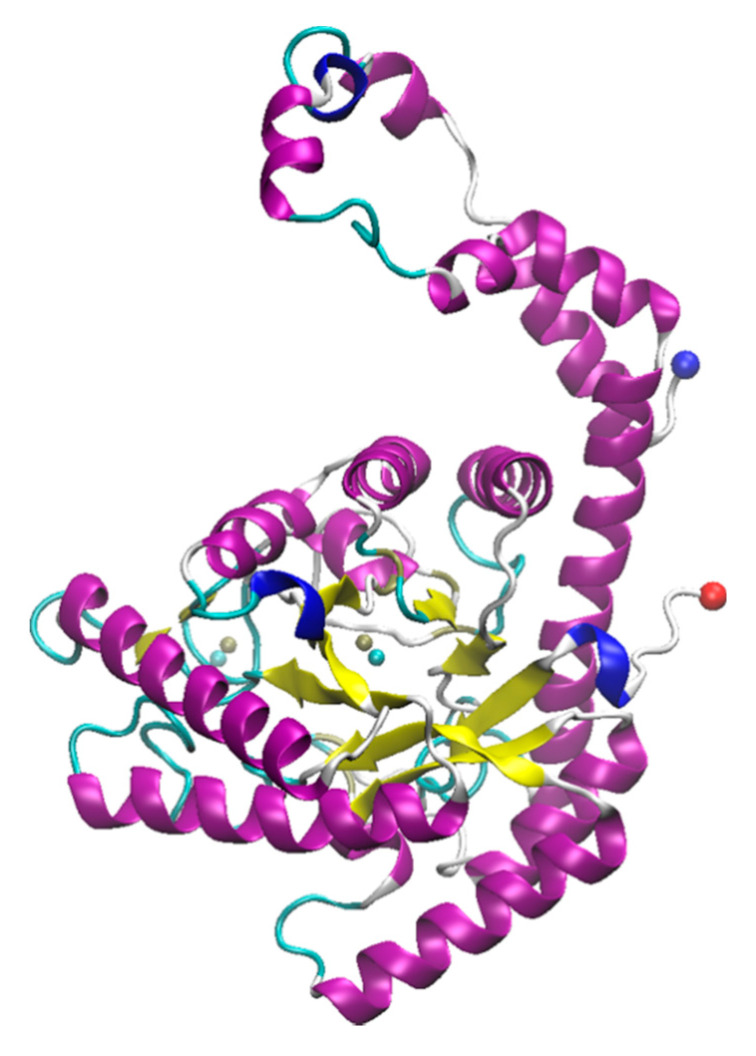
3D Structure of glucose isomerase from *Streptomyces rubiginosus*. The crystal structure was solved by Dauter and coworkers; downloaded from rcsb.org (1OAD) [145,199] The structure was visualized using VMD. The N- and C-termini are shown as red and blue spheres respectively while the manganase ions are shown in tan and the magnesium ions shown in cyan. The structure represents one monomer of a homodimer.

Glucose isomerase is a homotetrameric metalloenzyme with four catalytic centers and promiscuous functionality (Figure 6) [199]. The enzyme catalyzes reversible isomerizations of d-glucose to d-fructose as well as d-xylose to d-xylulose. Each of the catalytic centers has two subunits that form a pocket-like shape and have two divalent metal ion binding sites. Glucose isomerase is usually associated with metal ions like Mg^2+^, Co^2+^, or Mn^2+^, or a combination of these [200]. The active site contains the metal ions and several critical carboxyl containing residues (Asp and Glu) as well as a His residue involved in proton transfer. Glucose isomerase is a very important industrial enzyme for petroleum and food applications as it is used for production of ethanol for fuel as well as high fructose corn syrup [200]. One study compared effects of various ILs on the activity of glucose isomerase toward converting glucose to fructose [201]. This study investigated the ILs [DMEA][F], [DMEA][Pr], [DMEA][De], [Choline][Pr], [DMBA][Pr], [MPIP][Ac], [DBEA][Oc], [Choline][Ac], [EMIM][Ac], [EMIM][Cl], [BMIM][Cl] and [BMIM][Ac]. Among these ILs [EMIM][Cl] and [BMIM][Cl] showed a deactivating effect on the glucose isomerase and no fructose production was observed. On the other hand, [DBEA][Oc] showed the highest fructose production (of about 52%) in comparison to other ILs, when the final water content was kept at 21% *w/w*. In addition, [DBEA][Oc] was the only IL which was also able to produce mannose at 2% *w/w*, while all other ILs showed intermediate fructose production. These results indicate that the presence of ILs can significantly affect enzyme activity/stability and it is important to screen multiple ILs to find the one which provides optimum results [201].

ILs have also shown to impact the crystallization and X-ray diffraction resolution for glucose isomerase [202]. For instance, in a study by Judge et al., glucose isomerase was crystallized in presence of ILs such as [EMIM][BF_4_], [EMIM][Cl], [BMIM][Cl], [HMIM][Cl], triisobutyl (methyl) phosphonium p-toluenesulfonate, [n-BP][Cl]. Among all ILs the triisobutyl (methyl) phosphonium p-toluenesulfonate was shown to produce bigger crystals with a change in the morphology of glucose isomerase crystals compared to control samples without ILs [202]. However, proper optimization of the IL concentration during the crystallization is necessary because in some cases higher amounts of IL might negatively impact the crystal. For example, when crystallization of glucose isomerase was carried out with [BMIM][Cl] at 0 M, 0.2 M, and 0.4 M, plate-like crystals of glucose isomerase were obtained only with 0.2 M IL, while the samples with no IL gave salt precipitates and samples with 0.4 M IL did not yield any crystals or precipitates [203]. Furthermore, a synergistic effect was observed when ILs were combined with other techniques that also promote enzyme activity. For instance, the activity of immobilized glucose isomerase and reaction yield for glucose conversion to fructose was found to be highest when [EMIM][Cl] was used in combination of ultrasound irradiation, compared to use of only the IL or ultrasound irradiation individually [204].

## 3. Conclusions/Perspective

Depending on the physicochemical properties of ILs such as polarity, alkyl chain length in cation, anions in IL, hydrophobicity, and viscosity, ILs can have differential effects on protein stability. Some ILs have been shown to improve the stability of proteins, some are inert, and others disruptive to protein structure and function. Because of these unique properties, ILs have applications in multiple fields such as chemistry/synthesis, biotech, pharmaceutical, and the electronics industries. Specifically, ILs that have been shown to stabilize proteins can potentially be beneficial in developing formulations of protein therapeutics or in industrial processes using biocatalysts.

As the protein stabilization or destabilization is very specific to the chemistry of ILs, a rational selection of IL for protein under investigation is necessary before using it as a solvent for improving protein stability or activity. There is only limited knowledge regarding the mechanism of protein stabilization or destabilization in the presence of ILs and therefore research is still needed to understand fundamental chemistry of ILs and how they interact with proteins. This is a crucial step before ILs can be effectively incorporated into protein production, purification, or biocatalytic processes. These experiments, in total, should aim to develop a predictive model for IL-biomolecule systems which varies both the cation and anion of the IL based on the properties and functional environment of the protein. This is a critical but challenging process because of the variability in IL compositions, ongoing development of new ILs, and the variability and complexity between different proteins.

One approach which has been recently described is instead of single entities, mixtures of different ILs have also been used for obtaining better protein stability [205]. In addition to experimental approaches for evaluating the effect on ILs on the protein stability, various in silico analyses have also been performed. For instance, a study using molecular dynamics simulation analysis indicated that in the presence of ILs the bovine serum albumin does not destabilize the structure it adopts, which was also confirmed by experimental analysis [206]. These molecular dynamics simulations will undoubtedly help to narrow the field of potential IL candidates for specific protein and biomolecular applications.

Importantly, in the study of metalloproteins with ILs, there are still numerous questions regarding mechanism of IL-protein interactions. Most importantly, the majority of studies focus on the protein structure for obvious reasons. However, it leaves any direct interactions between ILs and the metal ions ambiguous. While in most cases it is clear from spectroscopic measurements that the metal ions are no longer properly coordinated in the protein structure, which was the initial driving force? Do IL interactions directly with the metal cause a destabilization in the protein or does destabilization of the protein cause a loss or displacement of the metal ion from the native binding site? While the latter is intuitive, there is only preliminary direct evidence. Additional studies that directly interrogate the metal sites such as vibrational methods and magnetic circular dichroism will help shed light on this question.

Another important aspect that must be considered when discussing IL-biomolecule applications is toxicity. The ability of a specific IL to stabilize a protein structure does not inherently mean it will be stabilizing to all proteins and may cause cytotoxic effects through other mechanisms. Similarly, there is no guarantee that because an IL is well tolerated by one organism that it will be equally biocompatible with all organisms. As such, the study of IL toxicity is an ongoing and rich area of research with numerous groups focused on this problem. Many studies have shown that some ILs can exhibit environmental toxicity or organismal cytotoxicity [207,208,209]. Alternatively, there are numerous examples in the literature of ILs that exhibit low levels of cytotoxicity, encouraging the investigation of these formulations for biological and pharmaceutical applications [207,208,210,211,212,213]. Our own work has shown that the cytotoxicity of ILs with imidazolium-based cations is dependent on alkyl chain length but can be used synergistically with traditional antimicrobials well below the cytotoxicity window against human cells [214,215]. These findings parallel that of many other groups which have shown a link between lipophilicity and cytotoxicity for ILs [216,217]. However, in light of the vast number of IL species combined with the breadth of biological species, it is necessary to expand the throughput of screening IL toxicity. Many groups have employed computational QSAR approaches to build predictive models of IL toxicity to cells [216,218,219,220]. These studies can potentially yield a great deal of insight for experimentalists in the design of IL formulations for specific applications.

Finally, the significance and importance of metalloproteins will continue to grow. Numerous industrial processes rely on metalloproteins for catalysis. These include enzymes such as metalloproteases, laccases, cellulases, lipases, phosphatases, and amylases [221,222]. Further, some of the metalloproteins are involved in the progression of the cancer and other diseases [223]. Once suitable ILs are identified and their effects on a given protein have been thoroughly evaluated, they can be successfully be used in combination with those targets to enhance or reduce activity. Because of having these beneficial properties, ILs have potential to serve as an ideal vehicle for protein therapeutics, a combinatorial therapeutic component, and an activity-enhancing additive in industrial processes in the near future.

## Figures and Tables

**Table 1:** Summary of studies done pertaining to monitoring the effects of ionic liquids (ILs) on laccase.

Laccase Source	IL	Structure	Study Done	Results	Ref
*Aspergillus*	1-ethyl-3-methylimidazolium ethylsulfate ([EMIM][EtSO_4_])	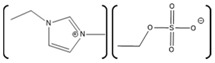	Activity at different temperatures in the presence or absence of ILs	([EMIM][EtSO_4_] IL decrease the activity of laccase	[154]
*Trametes versicolor*	tetramethylammonium trifluoromethanesulfonate ([TMA][TfO]).	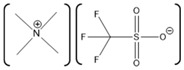	Enzyme kinetics, time-dependent fluorescence, CD analysis	[TMA][TfO] can stabilize laccase and keep its catalytic efficiency unchanged.	[155]
*Trametes versicolor*	1-butyl-3-methylimidazolium trifluoromethanesulfonate ([BMIM][TfO]), 1-butyl-1-methylpyrrolidinium trifluoromethanesulfonate ([BMPyr][TfO]	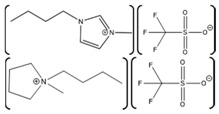	Enzyme kinetics, Time dependent fluorescence, CD analysis	High level of [BMIM][TfO] or [BMPyr][TfO] destabilizes laccase and decrease its activity	[155]
*Trametes versicolor*	1-butyl-3-methylimidazolium chloride, [BMIM]Cl; 1-ethyl-3-methylimidazolium ethylsulfate, [EMIM] [EtSO_4_]	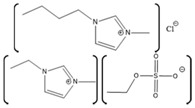	Enzyme kinetics (spectrophotometric measurement of activity at 420 nm)	Inhibition of laccase activity	[156]
*Trametes versicolor*	Pyrrolidinium Formate ([Pyrr][F]); the Morpholinium F ([morph][F]), mb b (C_5_H_11_NO_3_)	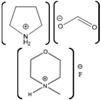	Fluorescence	Red shift in emission maximum in the presence of ILs	[157]
*Trametes versicolor*	1-butyl-3-methylimidazolium methyl sulfate, [BMIM][MeSO_4_] and 1,3-dimethylimidazolium methyl sulfate, [MvMIM][MeSO_4_],	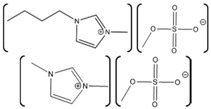	Effect of both water soluble ILs at different concentrations monitored using laccase activity assay	Laccase activity did not chang up to 25% IL concentration in both cases. However, at 35% both the ILs increased the laccase activity ~1.7 times	[158]
*Trametes versicolor,*	Choline dihydrogen phosphate [Chol][H_2_PO_4_]	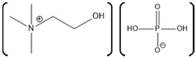	Fourier Transform Infra-Red spectroscopy (FT-IR)	Effective in increasing and stabilizing laccase activity	[122]
*Bacillus HR03*	1-ethyl-3-methyl imidazolium chloride [EMIM][Cl]; butyl-3-methyl imidazolium chloride [BMIM][Cl]; hexyl-3-methyl imidazolium chloride [HMIM][Cl]	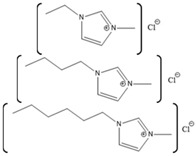	Enzyme activity, fluorescence, CD	As IL concentration increases, activity decreases. (Km increases)	[159]

**Table 2:** Summary of studies done pertaining to monitoring the effects of ILs on myoglobin.

Myoglobin Source	IL	Structure	Study Done	Results	Ref
Horse-heart myoglobin	1-ethyl-3-methylimidazolium phenylalanine [EMIM][Phe]	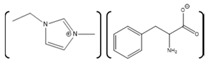	Fluorescence and circular dichroism spectroscopy	Small concentrations of [EMIM][Phe] increase helicity and stabilize protein, while higher concentrations lead to increase in beta structures	[174]
Horse-skeletal myoglobin	1-butyl-3-methyl imidazolium tetrafluoroborate ([BMIM][BF_4_]); 1-butyl-3-methyl pyrrolidinium tetrafluoroborate ([Pyrr][BF_4_]); 1-ethyl-3-methylimidazolium acetate [EMIM][Ac]); Tetramethyl guanidinium acetate [TMG][Ac])	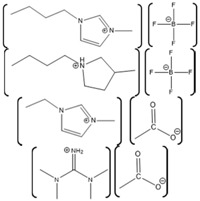	Temperature stability studies, hydrogen deuterium exchange (HDX) experments, unfolding kinetics	ILs enhances myoglobin unfolding kinetics	[123]
Horse-skeletal myoglobin	Tetramethyl ammonium hydroxide [TMA][OH]; Tetraethyl ammonium hydroxide [TEA] [OH]; Tetrapropyl ammonium hydroxide [TPA] [OH]; Tetrabutyl ammonium hydroxide [TBA] [OH]	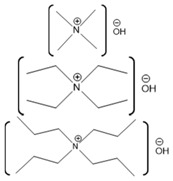 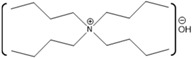	Fluorescence and circular dichroism (CD)	Decreases thermal stability of myoglobin	[175]
Salt free myoglobin (Mb)	1-butyl-3-methylimidazolium cation [BMIM]+	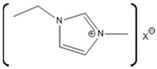 X = SCN^−^, HSO_4_^−^, Cl^−^, Br^−^, CH_3_COO^−^ and I^−^	UV-vis spectroscopy, fluorescence spectroscopy, CD	A negative impact on the stability of Myoglobin, a sharp decrease in the transition temperature (Tm) of the myoglobin	[176]
Horse- skeletal myoglobin	1-butyl-3-methylimidazolium chloride ([BMIM]Cl); 1-ethyl-3-methylimidazolium acetate ([EMIM]Ac); 1-butyl-3-methylimidazolium tetrafluoroborate ([BMIM][BF_4_])	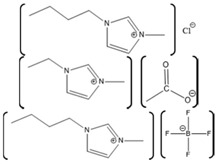	Detergent (*N*,*N*-dimethyl-*N*-dodecylglycine betaine) induced denaturation and heme-loss from myoglobin monitored by fluorescence and circular dichroism	ILs have no significant effect on heme dissociation as well as denaturation of myoglobin	[177]
Horse-skeletal myoglobin	Ethylmethylimidazolium acetate ([EMIM]Ac) and Butylmethylimidazolium boron tetrafluoride ([BMIM][BF_4_])	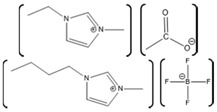	Guanidin HCl induced myoglobin unfolding by combined absorption/fluorescence spectroscopic	[EMIM]Ac does not affect myoglobin unfolding (up to 150 mM), while [BMIM][BF_4_] facilitated myoglobin unfolding	

**Table 3:** Effect of various ILs on the melting temperature from fluorescence and differential scanning calorimetry (DSC) along with secondary structure composition of myoglobin determined from Far-UV CD spectra (adapted from reference [172]).

Sample	Fluorescence *T*_m_ (°C)	DSC *T*_m_ (°C)	α-Helix (%)	β-Strand (%)
Buffer	65.1	67.0	56.12	7.77
[TEA][P]	87.1	86.8	69.92	1.57
[DEA][P]	84.0	78.9	64.05	3.11
[TMA][P]	83.1	77.9	61.13	4.23
[TEA][SO_4_]	76.0	75.8	60.13	5.23
[DEA][SO_4_]	74.2	73.4	58.76	4.38
[TMA][SO_4_]	73.0	75.8	57.12	6.77
[TEA][Ac]	56.3	62.4	53.52	8.87
[DEA][Ac]	54.2	56.7	32.42	22.82
[TMA][Ac]	52.0	54.5	30.62	25.96
Urea (1 M)	NA	NA	54.63	7.23

NA = data not available.

**Table 4:** Summary of studies done pertaining to monitoring the effects of ILs on azurin.

Azurin Source	IL	Structure	Study Done	Results	Ref
Azurin from *P. aeruginosa*	1-ethyl-3-methylimidazolium chloride, [EMIM][Cl] 1-butyl-3-methylimidazolium chloride, [BMIM][Cl] 1-hexyl-3-methylimidazolium chloride, [HMIM][Cl] 1-octyl-3-methylimidazolium chloride, [OMIM][Cl]	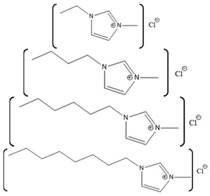	Temperature-dependent fluorescence and IR Spectroscopy, IR and VCD Spectroscopy temperature jump kinetics	ILs affected the protein structure by destabilizing it; however, the degree to which the protein unfolded is dependent on the ionic liquid in terms of hydrophobicity and alkyl chain length.	[174]
Azurin II was purified and expressed from *Alcaligenes Xylosoxidans* (Az). Pseudoazurin was isolated from *Achromobacter cyclastes* IAM 1013 (Paz).	Hydrated choline dihydrogen phosphate, [Chol][Dhp].	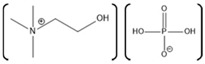	CD, Raman spectroscopy, enzyme activity	The protein and IL did not have an interaction that caused a disturbance in the structure or function of the protein.	[123]
Azurin from *P. aeruginosa*	Hydrated choline dihydrogen phosphate [Chol][Dhp].	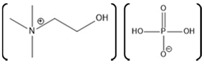	Raman spectroscopy, direct electrochemistry of azurin performed on self-assembled monolayer (SAM)-AuNP Electrode	The protein maintained its structure and its active site in the presence of the IL. Fast and stable electron transfer reactions could occur over a range of temperature values at longer periods of time.	[175,185]

## Abbreviations

IL	cations
[EMIM]	1-ethyl-3-methylimidazolium
[BMIM]	1-butyl-3-methylimidazolium
[HMIM]	1-hexyl-3-methylimidazolium
[MMIM]	1,3-dimethylimidazolium
[BzMIM]	1-benzyl-3-methylimidazolium
[OMIM]	1-octyl-3-methylimidazolium
[PMIM]	1-propyl-3-methylimidazolium
[BBIM]	1,3-dibutylimidazolium
[BMPyr]	1-butyl-3-methylpyrrolidinium
[OH-EMIM]	1-(2-hydroxyethyl)-3-methylimidazolium
[Me4N]	Tetramethyl ammonium
[Et4N]	Tetraethyl ammonium
[Pr4N]	Tetrapropyl ammonium
[Bu4N]	Tetrabutyl ammonium
[Me3NH]	Trimethyl ammonium
[Et3NH]	Triethyl ammonium
[Bu3NH]	Tributyl ammonium
[MTOA]	Methyl trioctyl ammonium
[BTMA]	Butyl trimethyl ammonium
[Chol]	Choline
[Gua]	guanidinium
[DMEA]	*N*,*N*-dimethylethanolammonium formate
[DMBA]	*N*,*N*-Dimethylbutylammonium propionate
[MPIP]	*N*-Methylpiperidinium acetate
[n-BP]	n-butylpyridinium
[DBEA]	*N*,*N*-dibutylethanolammonium
IL	anions
[BF_4_]	Tetrafluoroborate
[PF_6_]	Hexafluorophosphate
[Tf2N]	Bis(trifluromethane)sulfonimide
[Dmp]	Dimethyl phosphate
[MDEGSO_4_]	2-(2-methoxyethoxy) ethyl phosphate
[MeSO_3_]	Methyl sulfonate
[EtSO_4_]	Ethyl sulfate
[CF3OO]	Trifluoro acetate
[Dca]	Dicyanamide
[Dhp]	Dihydrogen phosphate
[OTf]	Trifluoromethane sulfonate
[MeSO4]	Methyl sulfate
[SO_4_]	Sulfate
[TMA]	Trimethyl acetate
[F]	Formate
[Pr]	Propionate
[De]	Deconate,
[Oc]	Octanoate
[Cl]	Chloride
